# LPS from* P. gingivalis* Negatively Alters Gingival Cell Mitochondrial Bioenergetics

**DOI:** 10.1155/2017/2697210

**Published:** 2017-05-16

**Authors:** Kiran Napa, Andrea C. Baeder, Jeffrey E. Witt, Sarah T. Rayburn, Madison G. Miller, Blake W. Dallon, Jonathan L. Gibbs, Shalene H. Wilcox, Duane R. Winden, Jared H. Smith, Paul R. Reynolds, Benjamin T. Bikman

**Affiliations:** ^1^College of Dental Medicine, Roseman University of Health Sciences, South Jordan, UT, USA; ^2^Department of Physiology and Developmental Biology, Brigham Young University, Provo, UT 84602, USA

## Abstract

**Objective:**

Oral inflammatory pathologies are linked to increased oxidative stress, thereby partly explaining their relevance in the etiology of systemic disorders. The purpose of this work was to determine the degree to which LPS from* Porphyromonas gingivalis*, the primary pathogen related to oral inflammation, altered gingival mitochondrial function and reactive oxygen species generation.

**Methods:**

Human gingival fibroblast (HGF-1) cells were treated with lipopolysaccharide of* P. gingivalis. *Mitochondrial function was determined via high-resolution respirometry.

**P. gingivalis:**

Mitochondrial function was determined via high-resolution respirometry.

**Results:**

LPS-treated HGF-1 cells had significantly higher mitochondrial complex IV and higher rates of mitochondrial respiration. However, this failed to translate into greater ATP production, as ATP production was paradoxically diminished with LPS treatment. Nevertheless, production of the reactive H_2_O_2_ was elevated with LPS treatment.

**Conclusions:**

LPS elicits an increase in gingival cell mitochondria content, with a subsequent increase in reactive oxygen species production (i.e., H_2_O_2_), despite a paradoxical reduction in ATP generation. These findings provide an insight into the nature of oxidative stress in oral inflammatory pathologies.

## 1. Introduction

In the United States, one out of every two American adults over the age of 30 has periodontal disease [1]. Periodontal disease manifests in two forms: gingivitis and periodontitis. While gingivitis is the inflammation of the gingival tissue, without the loss of supporting tooth structure, periodontitis, the more severe form of periodontal disease, is an infectious disease characterized by inflammation within the supporting tissues of the teeth with progressive attachment and bone loss [2]. Chronic periodontitis is the most common form of periodontitis, initiated and sustained by the presence of microorganisms, the innate immune response, and a variety of genetic factors [3].

Understandably, the primary focus for periodontal disease is the oral cavity, though its consequences extend systemically, including insulin resistance [4], diabetes [4, 5], arthritis [6], and heart disease [7]. Research efforts have largely focused on inflammation as the major mediating mechanism between periodontitis and systemic complications [8]. However, in addition to inflammation, alterations in mitochondrial function leading to oxidative stress have also been implicated in mediating both oral and systemic pathologies [9–11], though this paradigm requires further exploration.

Like other cells, oral gingiva is highly responsive to the bacterial endotoxin, lipopolysaccharide (LPS), which is prevalent in periodontal disease [12]. Indeed, LPS may be responsible for the inflammation and oxidative stress that accompany the periodontal disease. Considering the relevance of oxidative stress in a myriad of oral and systemic pathologies, we sought to better understand the concise consequences of LPS on gingival cell mitochondrial bioenergetics.

## 2. Methods

### 2.1. Cell Culture

Human gingival fibroblast (HGF-1) cells were maintained in Dulbecco's modified Eagle's medium (DMEM) plus 15% fetal bovine serum (FBS) and 2% Pen/Strep at 37°C. The culture medium was replaced twice a week until the cells reached 80% confluency. The cells were rinsed with phosphate-buffered saline (PBS), trypsinized, and split into 6–10 mm plates. The medium was replaced twice a week until the cells reached about 80% confluency. For LPS treatment, cells were treated with lipopolysaccharide of* Porphyromonas gingivalis* (LPS, 5 *μ*g/mL) for 48 h.

### 2.2. Mitochondrial Respiration

Cells were prepared for mitochondrial respiration as described previously [13, 14] before being transferred to respirometer chambers using the Oroboros O2K oxygraph. Electron flow through complex I was supported by glutamate  +  malate (10 and 2 mM, resp.) to determine leak oxygen consumption (GM_*L*_). Following stabilization, ADP (2.5 mM) was added to determine oxidative phosphorylation capacity (GM_*P*_). Succinate was added (GMS_*P*_) for complex I  +  II electron flow into the Q-junction. To determine full electron transport system capacity in cells over oxidative phosphorylation, the chemical uncoupler carbonyl cyanide 4-(trifluoromethoxy)phenylhydrazone (FCCP) was added (0.05 *μ*M, followed by 0.025 *μ*M steps until maximal O_2_ flux was reached; GMS_*F*_). Mitochondrial membrane integrity was tested in all experiments by adding cytochrome* c* (not shown; 10 *μ*M). Lastly, residual oxygen consumption was measured by adding antimycin A (2.5 *μ*M) to block complex III action, effectively stopping any electron flow, which provides a baseline rate of respiration. Following the respiration protocol, samples were removed from the chambers and used for further analysis, including protein quantification. Respiratory control ratio (RCR) and uncoupling control ratio (UCR) were determined by the ratio of GM_*P*_ : GM_*L*_ and GMS_*F*_ : GMS_*P*_, respectively.

### 2.3. ATP Production

ATP production was determined using the Oroboros O2K oxygraph, as described previously [15]. Briefly, ATP production was determined after addition of ADP (2.5 mM) by measuring changes in free extramitochondrial [Mg^+^] using Magnesium Green (MgG; Life Technologies). Fluorescence was measured with 503 nm and 530 nm excitation and emission, respectively.

### 2.4. H_2_O_2_ Production

An average rate of H_2_O_2_ generation was determined using the Oroboros O2K oxygraph. Amplex UltraRed (5 *μ*M; Thermo Fisher), horseradish peroxidase (1 U/mL; Sigma), and superoxide dismutase (5 U/mL; Sigma) were added to the chambers prior to the addition of cells. Following addition of various substrates indicated above, H_2_O_2_ (0.1 *μ*M; Sigma) was added as a calibration to determine endogenous H_2_O_2_ production.

### 2.5. Mitochondrial Morphology

HGF-1 cells were grown to confluence in chamber slides (NUNC Lab-Tek II Chambered Coverglass System; 155382). The mitochondrial dye MitoTracker Red CMXRos (Molecular Probes; M7512), dissolved in anhydrous dimethylsulfoxide (DMSO), was added to the culture at 250 nM. The cells were incubated for 30 minutes at 37°C in the dark and then visualized using a confocal microscope (Olympus IX81).

### 2.6. Statistical Methods

Data are presented as means ± SEM. Data were compared with one-way ANOVA or Student's *t*-test (Graphpad Prism; Microsoft Excel). Significance was set at *P* < 0.05.

## 3. Results

### 3.1. LPS Increases Mitochondrial Electron Transport Complex IV and Respiration

**Figure 1:**
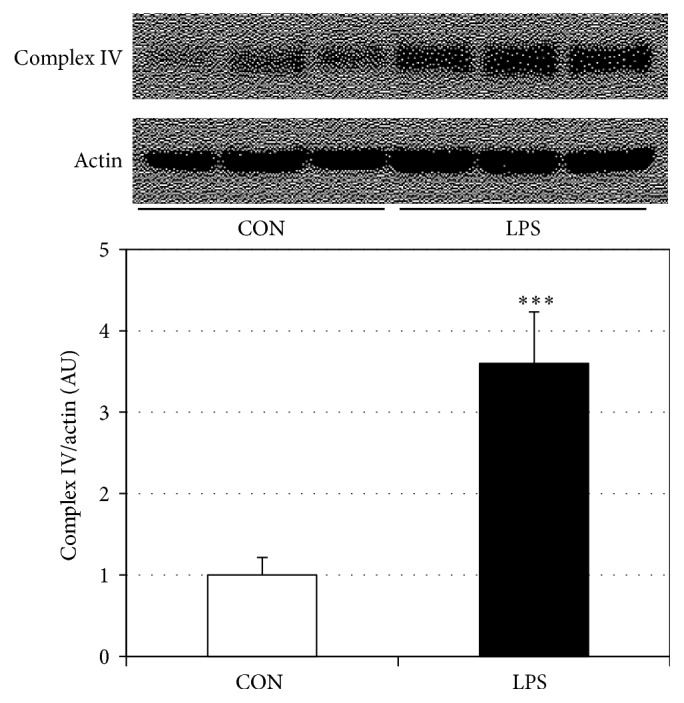
LPS increases complex IV protein expression in HGF-1 cells. Human gingival fibroblasts were treated with LPS (5 *μ*g/mL) for 48 h, after which complex IV protein levels were determined (*n* = 4). ^*∗∗∗*^*P* < 0.001.

**Figure 2:**
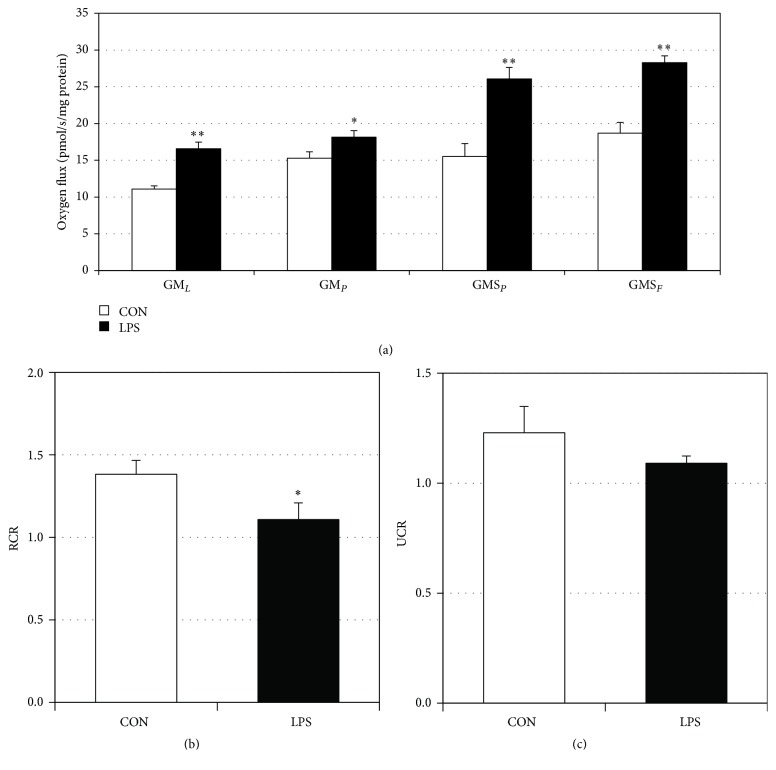
LPS alters HGF-1 mitochondrial bioenergetics. Mitochondrial assessments were determined in permeabilized (digitonin, 1 mg/mL) HGF-1 cells following treatment with LPS (5 *μ*g/mL) for 48 h. To measure mitochondrial respiration (a), samples were sequentially treated with GM_*L*_ (glutamate (10 mM) + malate (2 mM)), GMP_*P*_ (glutamate (10 mM) + ADP (2.5 mM)), GMS_*P*_ (glutamate (10 mM) + succinate (10 mM)), and GMS_*F*_ (glutamate (10 mM) + FCCP (0.05 *μ*M)). Respiratory control ratio (RCR, (b)) and uncoupling control ratio (UCR, (c)) were determined by the analysis indicated in the Methods (*n* = 4). ^*∗*^*P* < 0.05; ^*∗∗*^*P* < 0.01.

Human gingival fibroblasts incubated with LPS from* P. gingivalis* had an over threefold increase in complex IV protein content (Figure 1), consistent with previous reports (Hansen et al., 2015). Moreover, independent of the protein amount, LPS-treated cells had significantly increased respiration rates at each step of the respiration protocol (Figure 2(a)). However, minimal differences were noted between respiration rates in the LPS-treated cells in the leak state (GM_*L*_) and ADP addition (GM_*P*_) and succinate addition (GMS_*P*_) and FCCP uncoupler (GMS_*F*_), respectively. When comparing post hoc analyses of mitochondrial respiration, we found that LPS-treated gingival cells experienced a reduction in the respiratory control ratio (Figure 2(b)), a rough indication of mitochondrial health, and the uncoupling control ratio was decreased in LPS-treated gingival cells compared to control (Figure 2(c)).

### 3.2. LPS Decreases ATP Production and Increases H_2_O_2_ Production

**Figure 3:**
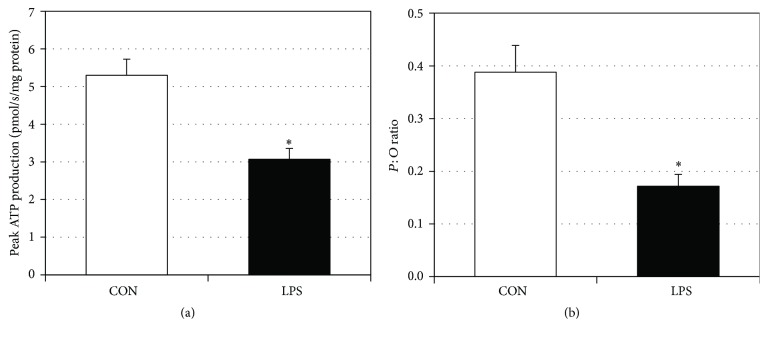
LPS reduces ATP production in HGF-1 cells. To determine ATP production following LPS treatment ((a), 5 *μ*g/mL for 48 h), Magnesium Green fluorescence (503/530 nM) was determined during respiration after calibration with ADP (2.5 mM) (*n* = 3). The rate of peak ATP production was then compared against the rate of oxygen consumption to determine the P : O ratio ((b), *n* = 3). ^*∗*^*P* < 0.05.

**Figure 4:**
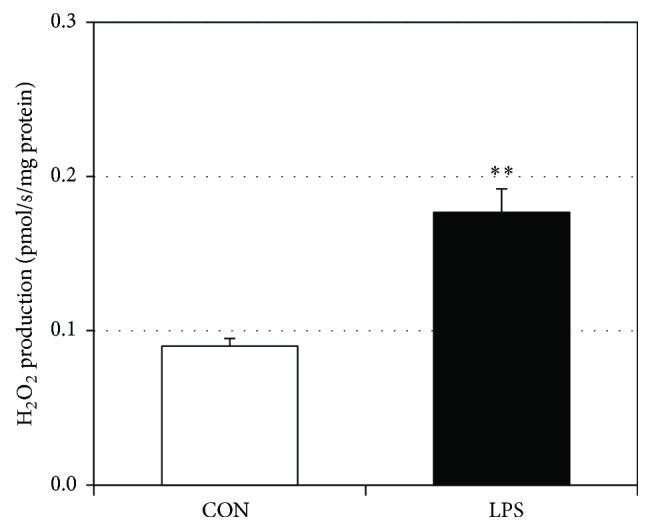
LPS increases H_2_O_2_ production in HGF-1 cells. To determine H_2_O_2_ production following LPS treatment ((a), 5 *μ*g/mL for 48 h), Amplex UltraRed fluorescence (530/590 nM) was determined during respiration after calibration with H_2_O_2_ (0.1 *μ*M, *n* = 3). ^*∗∗*^*P* < 0.01.

In addition to general respiration, we analyzed specific use of oxygen by measuring ATP production and H_2_O_2_ generation. LPS-treated gingival fibroblasts had a significant reduction in ATP production (Figure 3(a)). Moreover, the ratio of peak ATP production to oxygen consumption decreased in the LPS-treated gingival cells compared to control (Figure 3(b)), suggesting a deleterious change in mitochondrial physiology. Lastly, H_2_O_2_ production from LPS-treated gingival fibroblasts was significantly higher than in control (Figure 4), indicating increased oxidative stress.

### 3.3. LPS Increases Gingival Cell Mitochondrial Fission

**Figure 5:**
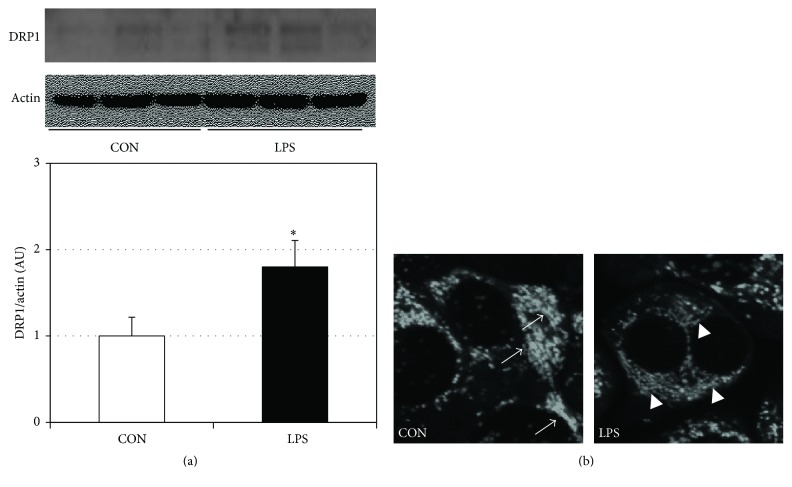
LPS increases mitochondrial fission in HGF-1 cells. Following LPS treatment (5 *μ*g/mL for 48 h), cells were either harvested to determine DRP1 levels via western blot ((a), *n* = 4) or visualized via confocal microscopy ((b); white arrows indicate sections of fusion; white arrowheads indicate fission; *n* = 3). ^*∗*^*P* < 0.05 for CON versus LPS.

We have previously found that LPS forces sustained mitochondrial fission in muscle cells and that fission is a key event in disrupting mitochondrial physiology [16, 17]. We found that the same series of events occurs in gingival cells. In particular, we observed a significant increase in protein levels of dynamin-related protein 1 (DRP1) following LPS treatment (Figure 5(a)). DRP1 is a key regulator of mitochondrial fission. This finding was substantiated by visualizing a greater degree of segmented and distinct mitochondria following LPS treatment (Figure 5(b)).

## 4. Discussion

The oral cavity contains a remarkable number of diverse bacteria. Of this substantial cohort, roughly 400 are thought to predominate during periodontal infections [18]; and of this group, a substantial portion contain lipopolysaccharides (LPS) in the cell wall. LPS is a powerful ligand that activates multiple immune-related receptors and, not surprisingly, has potent immune-activating effects on white blood cells, including macrophages and lymphocytes. In addition to classically promoting cytokine production, LPS also alters mitochondrial function in these cells [19]. However, the full extent to which LPS alters gingival mitochondrial function remains unclear.

The purpose of these studies was to better understand the pathology of gingival disease, with particular emphasis on the altered mitochondrial bioenergetics in the presence of LPS. We found that LPS elicited potent pathogenic changes in gingival cell mitochondrial function. Importantly, for the first time, we found that gingival cell LPS treatment yielded a significant increase in mitochondrial respiration while* reducing* ATP production and* increasing* H_2_O_2_ generation. These two changes in particular, namely, reduced ATP generation and increased H_2_O_2_ production, represent fundamental changes in mitochondrial homeostasis that would readily yield decayed cellular function. Furthermore, these changes may arise from the LPS-induced alteration in mitochondrial morphology, namely, forced mitochondrial fission, likely via increased DRP1 expression. Importantly, these are highly novel findings that, when considered in combination, allow a greater appreciation of the extent of gingival cell mitochondrial changes with LPS exposure. For example, Bullon et al. [20] found increased reactive oxygen species (ROS) production in gingival cells after LPS treatment, despite overall reduced mitochondrial function. However, there was no measurement of ATP.

Collectively, our data contribute to a growing paradigm regarding the nature of the pathogenic mitochondrial changes that occur in gingival cells with increased LPS exposure. The combination of a reduction in ATP generation and enhanced H_2_O_2_ production reveals an interesting shift in oxygen use. While oxygen consumption was increased with LPS treatment, it is evident that, rather than being appropriately used to facilitate ATP production via the electron transport system, which is necessary to maintain cellular homeostasis, oxygen is rather contributing to the formation of reactive oxygen species.

Importantly, the relevance of increased ROS generation arising from gingival inflammation extends well beyond the oral cavity. While certainly relevant in oral pathologies, including destruction of oral cells [21], the oxidative stress arising from increased LPS exposure to gingival cells may play a role in multiple systemic disorders. In particular, the ROS spread into the general circulation and increase the risk of cardiovascular disorders [22, 23], stroke [24], insulin resistance [25], and even pregnancy complications [26].

In conclusion, these studies reveal multiple novel findings regarding the mitochondrial response within gingival cells to LPS from* P. gingivalis*. Overall rate of mitochondrial respiration is increased, despite an atypical and significant reduction in ATP generation. Rather than being used to produce ATP, the increased respiration appears to be increasing the production of harmful H_2_O_2_, which may drive oral and systemic pathologies. Altogether, these data suggest a potential role for antioxidant therapies in helping mitigate the consequences of orally derived oxidative stress.
